# Weekly frequency of school-based HIIT improves adiposity and fitness in children with obesity

**DOI:** 10.1016/j.isci.2026.115715

**Published:** 2026-04-11

**Authors:** Yuhang Gao, Meng Cao, Yan Xie, Yuanzhi Zhou, Ti Zhang, Chun Wang, Xiaodong Wang

**Affiliations:** 1 Faculty of Education, Shenzhen University, Shenzhen 518060, China; 2 School of Physical Education, Shenzhen University, Shenzhen 518060, China; 3 Faculty of Health and Wellness, City University of Macau, Macau, China; 4 Shenzhen Futian Third People’s Hospital, Shenzhen 518060, China; 5 Sports Teaching and Research Office, Huafu Experimental School, Shenzhen 518060, China; 6 Shenzhen Nanshan Center for Chronic Disease Control, Shenzhen 518060, China; 7 School of Humanities and Social Sciences, The Chinese University of Hong Kong, Shenzhen 518060, China; 8 Shenzhen Bao’an District Wangxiaodong Sports Rehabilitation Studio, Shenzhen 518060, China

**Keywords:** Health sciences, Medicine, Medical specialty, Obesity medicine

## Abstract

This 12-week, four-arm randomized controlled trial examined whether weekly frequency of school-based high-intensity interval training (HIIT) differentially affects adiposity and cardiorespiratory fitness in children with obesity. Sixty participants aged 10–12 years were randomized to a control group or HIIT performed 1, 2, or 3 times per week during physical education; 51 completed the study. Primary outcomes were body mass index (BMI)-z and 20 m shuttle run test (20mSRT) performance. BMI and BMI-z decreased over time, and HIIT performed at least twice weekly produced greater reductions than control. Waist-to-hip ratio also differed by group. All HIIT groups improved 20mSRT performance compared with the control. When expressed as 20mSRT-derived estimated VO_2_max, significant group and time effects were observed, although differences among HIIT frequencies were small. These findings suggest that once-weekly HIIT can improve aerobic fitness, whereas at least two sessions per week may be needed to optimize adiposity-related outcomes in children with obesity.

## Highlights

• School-based HIIT shows dose-dependent effects in children with obesity
• 1 session/week improves aerobic fitness; 2–3/week better for adiposity
• Once-weekly HIIT improves fitness whereas 2–3 sessions/week better improve adiposity
• Time-efficient, low-equipment protocols are feasible and safe in PE classes

## Graphical abstract

**Figure:**
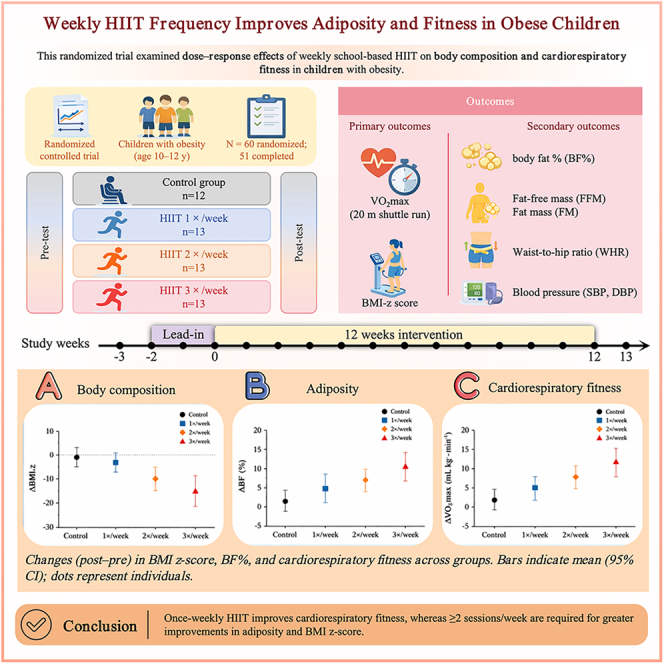

## Introduction

Childhood obesity remains a major global public health concern.^1^ By 2021, more than 170 million children worldwide were living with obesity, and prevalence is projected to rise further by 2050.^2^ In China, nearly one-fifth of youth aged 6–17 years are affected by overweight or obesity.^3^^,^^4^ Obesity in youth is a chronic condition associated with adverse cardiometabolic and psychosocial outcomes and often tracks into adulthood.^5^^,^^6^ Given the limited time available within school physical education (PE) curricula, identifying the minimum effective dose of HIIT and the optimal weekly frequency needed to elicit meaningful improvements is critical for scalable pediatric obesity interventions.^7^ Therefore, dose-frequency evidence in real-world school settings is needed to inform practical implementation.

High-intensity interval training (HIIT) consists of repeated bouts of vigorous exercise interspersed with recovery and is increasingly implemented in school- and community-based programs because it is time-efficient and well tolerated by youth.^8^^,^^9^ Beyond intensity, the weekly frequency of HIIT is a key component of exercise dose, shaping total training volume and the balance between training stimulus and recovery.^10^ Conceptually, increasing frequency could amplify cumulative energy expenditure and cardiometabolic adaptations, thereby improving adiposity-related outcomes (e.g., body mass index [BMI]-z, fat mass) and cardiorespiratory fitness (CRF)^11^^,^^12^; however, excessively frequent exposure may yield diminishing returns or compromise recovery, suggesting a potential threshold.^13^

In children with overweight/obesity, most HIIT interventions prescribe ∼3 sessions/week and generally report improvements in body composition and 20mSRT (20-meter shuttle run test) performance.^11^^,^^14^ However, direct head-to-head comparisons of different weekly frequencies using the same HIIT protocol are scarce.^15^ Emerging evidence in adults and non-obese youth suggests that lower-frequency HIIT (1–2 sessions/week) may still improve 20mSRT performance and some adiposity markers.^16^^,^^17^ However, whether similar benefits occur in children with obesity and whether adiposity and 20mSRT performance exhibit distinct frequency-response patterns remains unclear. Thus, defining the minimum effective weekly frequency and testing whether higher frequencies confer additional benefits are central to scalable school implementation and adherence.

Accordingly, this 12-week randomized trial investigated the dose-response effects of weekly HIIT frequency (1, 2, or 3 sessions/week versus a control group [CG]) on adiposity and CRF in children with obesity. The primary objectives were to determine whether BMI-z exhibits a frequency-dependent response and whether higher weekly frequencies lead to greater improvements in CRF. We hypothesized that 1 and 2 sessions per week would be as effective as 3 sessions, supporting the existence of a lower minimal effective dose.

## Results

Baseline characteristics were generally comparable across groups (Table 1). A chance imbalance in baseline 20mSRT was addressed by analysis of covariance (ANCOVA) models adjusting for baseline values. Across the 12-week intervention, exercise adherence exceeded 90% in all HIIT groups, no training-related injuries or adverse events were reported.

**Table 1:** Baseline characteristics of participants in the control and intervention groups, mean ± SD

Variable	CG (*n* = 12)	LFG (*n* = 13)	MFG (*n* = 13)	HFG (*n* = 13)
Boys/girls(n/n)	8/4	9/4	9/4	8/5
Tanner stage II (n, %)	12 (100)	13 (100)	13 (100)	13 (100)
BM (kg)	54.3 ± 2.7	54.7 ± 5.8	54.1 ± 6.8	55.6 ± 6.2
BMI (kg/m^2^)	23.8 ± 1.1	23.4 ± 2.5	23.8 ± 1.8	22.7 ± 0.6
BMI-z	2.9 ± 0.5	2.7 ± 1.2	2.9 ± 0.8	2.4 ± 0.4
BF% (%)	40.9 ± 4.2	41.5 ± 3.9	40.9 ± 5.0	41.8 ± 6.9
FM (kg)	22.2 ± 2.5	22.8 ± 3.7	22.3 ± 4.8	23.4 ± 5.5
FFM (kg)	18.7 ± 2.8	19.9 ± 3.3	19.0 ± 4.3	21.3 ± 5.3
VAT (cm^2^)	106.9 ± 30.3	97.8 ± 41.4	97.4 ± 37.9	102.4 ± 39.0
WHR	0.92 ± 0.05	0.90 ± 0.06	0.93 ± 0.05	0.87 ± 0.08
SBP (mmHg)	116.6 ± 6.8	114.9 ± 9.8	112.6 ± 7.0	115.4 ± 9.7
DBP (mmHg)	69.3 ± 8.1	68.9 ± 9.9	69.5 ± 4.0	67.5 ± 7.1
20-mSRT (laps)	19.4 ± 5.6	21.9 ± 4.2	25.6 ± 5.6	21.6 ± 4.9
Estimated VO_2_max (mL·kg^−1^·min^−1^)	38.0 ± 2.7	39.4 ± 2.5	40.4 ± 3.1	39.4 ± 2.0

BMI, body mass index; BF%, body fat percentage; FM, fat mass; FFM, fat-free mass; VAT, visceral adipose tissue; WHR, waist-to-hip ratio; SBP, systolic blood pressure; DBP, diastolic blood pressure; 20-mSRT, 20-m shuttle run test; estimated VO_2_max, 20-mSRT-derived estimate of maximal oxygen uptake. BMI-z was calculated using the World Health Organization (WHO) 2007 growth reference for school-aged children, based on the LMS method.

### Sex interaction analyses

Three-way repeated-measures ANOVA including sex as a between-subjects factor showed no significant group × time × sex interactions for the prespecified primary outcomes (BMI-z and 20mSRT performance; all *p* ≥ 0.88), indicating comparable improvements and frequency-response trends among boys and girls. Therefore, results are presented pooled across sex.

### Body composition

Repeated-measures ANOVA revealed significant time effects for BMI (*p* = 0.002, partial η^2^ = 0.092) and BMI-z (*p* = 0.002, partial η^2^ = 0.097), whereas BF% (*p* = 0.033, partial η^2^ = 0.046) and FM (*p* = 0.028, partial η^2^ = 0.049) did not reach the predefined significance threshold. No significant group × time interactions were observed for BMI, BMI-z, body fat percentage (BF%), fat mass (FM), visceral adipose tissue (VAT), or fat-free mass (FFM) (all *p* > 0.05).

ANCOVA adjusting for baseline values demonstrated that both moderate-frequency group (MFG) (2×/wk) and high-frequency group (HFG) (3×/wk) achieved significantly greater reductions in BMI and BMI-z compared with CG (all *p* < 0.05). Reductions in FM were numerically greater in the MFG and HFG than in the CG (nominal *p* < 0.05), whereas VAT changes (BIA-derived) were examined as exploratory and should be interpreted cautiously. For waist-to-hip ratio (WHR), a significant main effect of group was detected (*p* < 0.001, partial η^2^ = 0.185), whereas the time effect and interaction term were not significant (*p* > 0.05).

**Figure 1:**
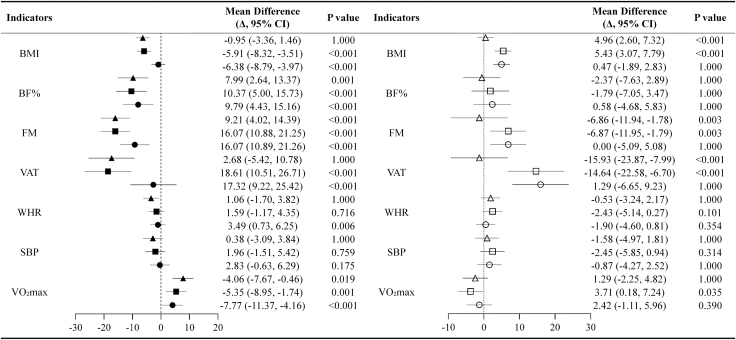
Forest plots of pairwise group differences in 12-week change (Δ) with 95% CIs Left: each intervention vs. control (● LFG–CG, ▪ MFG–CG, ▲ HFG–CG). Right: comparisons among intervention frequencies (○ LFG–MFG, □ LFG–HFG, Δ MFG–HFG). Outcomes shown: BMI (kg·m-2), BF% (%), FM (kg), VAT (cm2), WHR, SBP (mmHg), and estimated VO_2_max (mL·kg^−1^·min^−1^). Points indicate the mean difference in change from baseline between the two groups; horizontal bars are 95% confidence intervals. The dashed vertical line marks no between-group difference (Δ = 0). Negative values indicate that the first-named group decreased more (or increased less) than the comparator; positive values indicate the opposite. Sample sizes: CG = 12, LFG = 13, MFG = 13, HFG = 13.

Between HIIT subgroups, one-way ANOVA on change scores (Δ), followed by Bonferroni-adjusted post-hoc tests (adjusted α = 0.0125 for body composition outcomes), revealed that MFG and HFG achieved significantly greater reductions in BMI, BMI-z, and FM compared with LFG (all *p* < 0.0125). Differences in VAT (BIA-derived) were analyzed as exploratory and are interpreted cautiously; no significant differences were observed between MFG and HFG (*p* > 0.05). Notably, BF% and FM reductions in LFG were not significantly different from those in HFG (*p* > 0.0125), suggesting diminishing returns for certain adiposity outcomes beyond one session per week. Full pre- and post-intervention values are presented in Table 2. Figure 1 presents forest plots of pairwise group differences in Δ scores across primary and secondary outcomes, highlighting the between-group effects with 95% confidence intervals.

**Table 2:** Changes in cardiometabolic and fitness variables across groups before and after the intervention, mean ± SD

	CG(*n* = 12)			LFG(*n* = 13)			MFG(*n* = 13)			HFG(*n* = 13)			*p*-Value			Partial η^2^
	pre	post	Δ	pre	post	Δ	pre	post	Δ	pre	post	Δ	Group	Time	Inter-action	
BM (kg)	54.3 ± 2.7	54.7 ± 2.5	0.4 ± 1.2	54.7 ± 5.8	54.4 ± 5.4	−0.3 ± 1.2	54.1 ± 6.8	51.1 ± 5.9∗∗	−3.0 ± 1.3	55.6 ± 6.2	52.3 ± 5.8∗∗	−3.3 ± 1.4	0.407	0.153	0.515	0.991
BMI (kg/m^2^)	23.8 ± 1.1	23.6 ± 1.1	−0.2 ± 0.6	23.4 ± 2.5	22.9 ± 2.2∗∗	−0.5 ± 0.5	23.8 ± 1.8	22.2 ± 1.5∗∗	−1.6 ± 0.5	22.7 ± 0.6	21.1 ± 0.8∗∗	−1.6 ± 0.5^##,††,§^	0.007	0.002	0.242	0.995
BMI-z	2.9 ± 0.5	2.8 ± 0.5	−0.0 ± 0.1	2.7 ± 1.2	2.5 ± 1.1∗∗	−0.0 ± 0.2	2.9 ± 0.8	2.1 ± 0.7∗∗	−0.3 ± 0.1	2.4 ± 0.4	1.6 ± 0.5∗∗	−0.4 ± 0.1^##,††,§^	0.006	0.002	0.193	0.914
BF% (%)	40.9 ± 4.2	41.7 ± 4.2	0.9 ± 2.0	41.5 ± 3.9	39.2 ± 4.1∗∗	−2.4 ± 1.5	40.9 ± 5.0	37.5 ± 3.5∗∗	−3.5 ± 2.5	41.8 ± 6.9	38.6 ± 6.3∗∗	−3.2 ± 2.4	0.399	0.038	0.382	0.986
FM (kg)	22.2 ± 2.5	22.8 ± 2.6	0.7 ± 1.1	22.8 ± 3.7	21.4 ± 3.7∗∗	−1.4 ± 0.7	22.3 ± 4.8	19.2 ± 3.5∗∗	−3.0 ± 1.7	23.4 ± 5.5	20.2 ± 4.5∗∗	−3.1 ± 1.6	0.333	0.032	0.301	0.969
FFM (kg)	18.7 ± 2.8	19.2 ± 2.5∗	0.5 ± 0.7	19.9 ± 3.3	20.2 ± 3.1∗∗	0.3 ± 0.3	19.0 ± 4.3	19.6 ± 4.4	0.6 ± 1.2	21.3 ± 5.3	21.8 ± 5.3∗∗	0.5 ± 0.5^#,§^	0.101	0.746	0.988	0.963
VAT (cm^2^)	106.9 ± 30.3	107.7 ± 28.5	0.8 ± 5.9	97.8 ± 41.4	94.6 ± 36.1	−3.2 ± 8.9	97.4 ± 37.9	80.3 ± 30.6∗∗	−17.1 ± 9.3	102.4 ± 39.0	84.2 ± 26.9∗∗	−18.2 ± 13.3	0.593	0.169	0.683	0.894
WHR (×100)	91.6 ± 4.7	92.2 ± 4.7	0.6 ± 1.5	90.1 ± 5.5	89.7 ± 5.3	−0.4 ± 2.1	92.8 ± 5.0	91.9 ± 4.4	−0.9 ± 2.7^#^	87.2 ± 7.9	84.6 ± 6.1∗∗	−2.6 ± 2.7^##,††,§§^	0.000	0.472	0.774	0.996
SBP (mmHg)	116.6 ± 6.8	116.8 ± 6.2	0.3 ± 2.6	114.9 ± 9.8	114.7 ± 9.3	−0.2 ± 3.9	112.6 ± 7.0	110.6 ± 6.1	−2.0 ± 3.7	115.4 ± 9.7	112.3 ± 8.7∗	−3.1 ± 3.8	0.774	0.434	0.875	0.995
DBP (mmHg)	69.3 ± 8.1	68.9 ± 6.3	−0.3 ± 6.5	68.9 ± 9.9	68.8 ± 12.1	−0.1 ± 5.9	69.5 ± 4.0	67.3 ± 3.2∗	−2.2 ± 2.7	67.5 ± 7.1	65.8 ± 3.9	−1.6 ± 4.6	0.596	0.472	0.947	0.989
20-mSRT (laps)	19.4 ± 5.6	22.2 ± 5.5∗∗	2.8 ± 2.8	21.9 ± 4.2	29.4 ± 5.9∗∗	7.5 ± 4.8^##^	25.6 ± 5.6	33.0 ± 6.0∗∗	7.4 ± 2.6^##,†^	21.6 ± 4.9	31.4 ± 6.3∗∗	9.8 ± 4.2^##^	0.000	0.000	0.161	0.957
Estimated VO_2_max (mL·kg^−1^·min^−1^)	38.0 ± 2.7	39.1 ± 2.5∗∗	1.2 ± 1.0	39.4 ± 2.5	42.2 ± 2.7∗∗	2.8 ± 1.5^##^	40.4 ± 3.1	43.8 ± 2.9∗∗	3.4 ± 0.9^##,†^	39.4 ± 2.0	43.6 ± 2.6∗∗	4.3 ± 1.5^##^	0.000	0.000	0.212	0.996

CG, control group; LFG, low-frequency group; MFG, moderate-frequency group; HFG, high-frequency group; BMI, body mass index; BMI-z, number of standard deviations a participant’s BMI is from the age- and sex-specific mean of the reference population (calculated using WHO reference standards); BF%, body fat percentage; FM, fat mass; FFM, fat-free mass; VAT, visceral adipose tissue; WHR values are presented as ratio ×100 for readability; SBP, systolic blood pressure; DBP, diastolic blood pressure; estimated VO_2_max, 20-mSRT-derived estimate of maximal oxygen uptake; 20-mSRT, 20-m shuttle run test. Within-group paired *t* tests are descriptive (not multiplicity-adjusted); primary inference is based on RM-ANOVA and ANCOVA with prespecified Bonferroni thresholds.

Within-group differences (pre vs. post) were assessed using paired *t* tests: ∗*p* < 0.05, ∗∗*p* < 0.01. Between-group differences in change scores (Δ) were analyzed using one-way ANOVA with post-hoc comparisons versus CG: ^#^*p* < 0.05, ^##^*p* < 0.01; vs. LFG group: ^†^*p* < 0.05; vs. MFG group: ^§^*p* < 0.05. Polynomial regression analysis was used to evaluate dose-response relationships between HIIT frequency and changes in outcome variables.

Repeated-measures ANOVA was used to test time group and interaction effects.

### Cardiometabolic health and fitness

For 20mSRT performance, improvements were observed in all HIIT groups compared with the CG (see Table 2). When expressed as 20mSRT-derived (field-estimated) VO_2_max, significant main effects of group (*p* < 0.001, partial η^2^ = 0.204) and time (*p* < 0.001, partial η^2^ = 0.242) were observed, although the group × time interaction was not statistically significant (*p* = 0.129). Post hoc comparisons revealed significant improvements in all HIIT groups compared with the CG (all *p* < 0.05). However, there were no significant differences in estimated VO_2_max improvements between LFG and HFG (*p* > 0.05), and MFG improvements were broadly comparable to both, suggesting diminishing incremental gains from two to three sessions/week over 12 weeks.

For SBP and DBP, no significant effects of group, time, or group × time interaction were found (all *p* > 0.05). Percentage changes in cardiometabolic outcomes are depicted in Figure 1. Between-group comparisons for estimated VO_2_max at follow-up were derived from ANCOVA models adjusting for baseline values.

### Dose-frequency response patterns

Dose-response analyses were conducted *in* HIIT groups only (frequency = 1/2/3). A frequency-dependent trend was evident for BMI (R^2^ = 0.340) and BMI-z (R^2^ = 0.440). For BF% (R^2^ = 0.127), FM (R^2^ = 0.127), and FFM (R^2^ = 0.148), quadratic models provided a better fit than linear (ΔR^2^ > 0), indicating attenuated returns beyond once-weekly training. In the frequency-response analysis, estimated VO_2_max was positively associated with weekly HIIT frequency (linear fit *R*^2^ ≈ 0.63), whereas the marginal gain from two to three sessions/week was small, suggesting a threshold or diminishing returns over 12 weeks.

## Discussion

This randomized controlled trial provides, to our knowledge, the first structured comparison of once-, twice-, and thrice-weekly HIIT on body composition and aerobic fitness in Chinese children with obesity, including both sexes. Across 12 weeks, all HIIT frequencies elicited favorable changes in adiposity and CRF, although not all omnibus tests reached statistical significance. In line with the results, BMI showed a clear time effect (*p* = 0.002; partial η^2^ = 0.092) and larger adjusted reductions in the moderate- and high-frequency groups versus the CG. 20-mSRT performance improved, with corresponding increases in 20-mSRT-derived estimated VO_2_max (*p* < 0.001; partial η^2^ = 0.242) and a strong association with training frequency (R^2^ ≈ 0.63), yet pairwise contrasts suggested diminishing returns beyond once-weekly training over 12 weeks. No group × time × sex interactions were observed, indicating similar responsiveness in boys and girls.

### Body composition

Indicators of body composition, such as FM, BF%, and VAT estimate, play a critical role in determining metabolic health and the long-term risk of chronic diseases in children with obesity.^18^^,^^19^ Reducing excess fat, especially visceral fat, remains a key goal in childhood obesity management.^20^^,^^21^ In our study, all HIIT intervention groups showed reductions in BMI, FM, BF%, and VAT estimates; however, after applying the pre-specified multiplicity-control approach, only BMI demonstrated a clear statistically significant improvement. The magnitude of change generally increased with training frequency. Specifically, the moderate- and high-frequency groups achieved greater reductions in BMI than the low-frequency and CGs, supporting a frequency-response pattern for BMI-related outcomes. In contrast, changes in FM and BF% were directionally favorable but did not consistently meet the corrected significance threshold, and VAT (BIA-derived) was analyzed as an exploratory outcome; these adiposity-related findings should therefore be interpreted cautiously. This may partly reflect limited statistical power to detect small effects after multiplicity correction in a modest sample.

Notably, the ≈3 kg reduction in BIA-estimated fat mass observed in the moderate- and high-frequency groups may appear large relative to the energy expenditure of the supervised HIIT sessions alone. This apparent “energy discrepancy” likely reflects the combined influence of (i) short-term variability and measurement error in BIA-derived estimates, which are sensitive to hydration and glycogen status, and (ii) unmeasured changes in energy intake and total physical activity or sedentary time outside PE that can meaningfully shift energy balance over 12 weeks. Therefore, the magnitude of FM change should be interpreted cautiously, and future trials should incorporate DXA or imaging together with objective monitoring of diet and total physical activity (e.g., accelerometry) to clarify the determinants of fat mass change.

Similar outcomes were reported by Cao et al.,^22^^,^^23^ who observed marked decreases in fat mass and body fat percentage after a 12-week sprint interval training program implemented in schools for children with obesity. However, unlike Cao’s study, which tested only one training frequency, our research directly compared multiple HIIT frequencies. Additionally, it included a CG, providing new evidence on the dose-response effects of HIIT in this population.

Our results are also consistent with several recent randomized controlled trials and meta-analyses conducted internationally. For example, a meta-analysis by Costigan et al. demonstrated that HIIT interventions, regardless of specific protocol, consistently led to improvements in body composition among overweight and obese youth.^24^ However, most prior studies implemented a single, moderate-to-high frequency of HIIT (typically three times per week), and there is limited data examining the relative efficacy of lower versus higher frequencies within the same study design. Similarly, a study by Logan et al. in adolescents found that both two and four sessions of HIIT per week produced significant reductions in adiposity^25^ but did not systematically examine the optimal frequency. In our study, a plateau effect was not observed within the tested frequency range (1–3 times per week), but the similar magnitude of changes between the moderate- and high-frequency groups, especially for FM and VAT, suggests a possible threshold effect.

Some adult and general adolescent studies suggest that benefits may be non-linear with increasing HIIT frequency or volume; our data did not indicate clear additional gains from 3 vs. 2 sessions/week over 12 weeks, consistent with a possible threshold within the tested range.^26^^,^^27^^,^^28^ The observed frequency-related differences in BMI are likely attributable to several physiological mechanisms. Increased training frequency may result in higher total energy expenditure and more consistent stimulation of fat oxidation.^29^^,^^30^ Furthermore, regular HIIT is known to enhance metabolic adaptations,^31^ such as improved mitochondrial function and increased post-exercise fat oxidation. HIIT can also modulate hormones like catecholamines and growth hormone,^28^ which contribute to increased lipolysis and reduction of visceral fat. Additionally, the time-efficient and structured nature of HIIT may improve adherence, thereby supporting long-term improvements in body composition among children.

### CRF

Estimated VO_2_max derived from the 20-mSRT, a commonly used index of aerobic capacity in youth, strongly predicts cardiovascular health in young people. Among children with obesity, low aerobic fitness is linked to hypertension, insulin resistance, and metabolic syndrome, while improvements correspond to lower long-term mortality from cardiovascular and other causes.^32^^,^^33^^,^^34^ Therefore, enhancing CRF is a central objective for obesity interventions in this population.

Over 12 weeks, all HIIT groups improved 20mSRT performance, with greater gains in the moderate- and high-frequency groups compared with the low-frequency and control conditions. When expressed as 20mSRT-derived (field-estimated) VO_2_max, improvements showed a clear frequency-related gradient, although the incremental gain from two to three sessions/week was modest. Our findings indicate an overall positive frequency-response for VO_2_max, but the increment from two to three sessions/week was modest, indicating a potential dose threshold within 12 weeks. This pattern is consistent with rapid early central and peripheral adaptations followed by diminishing returns at higher weekly frequency. This dose-response effect was statistically robust (*p* < 0.001, R^2^ = 0.632) and consistent across boys and girls.

These findings are consistent with Logan et al., who identified a direct relationship between HIIT frequency and improvements in aerobic fitness in children.^25^ Our work not only corroborates their conclusions but also extends this evidence base to include Chinese children with obesity, thereby addressing an important gap in the existing literature. Whereas certain adult studies have documented plateaus or even adverse outcomes with excessively high HIIT frequencies, our data did not show such diminishing returns within the evaluated frequency range.^26^^,^^27^

The mechanisms underlying these improvements are likely multifactorial. Regular high-intensity exercise enhances stroke volume, cardiac output, and peripheral oxygen utilization, leading to superior adaptations in both central and peripheral components of the oxygen transport system.^35^ Additionally, HIIT promotes mitochondrial biogenesis and oxidative enzyme activity in skeletal muscle, further supporting aerobic capacity.^36^ Frequent HIIT may also improve endothelial function and autonomic regulation, contributing to overall cardiovascular health.

In children with obesity, once-weekly school-based HIIT is sufficient to improve 20mSRT performance, whereas ≥2 sessions/week appear necessary to optimize adiposity-related outcomes (e.g., BMI/BMI-z and WHR) over 12 weeks. Practically, schools may start with 1 session/week to maximize feasibility and uptake, then progress to 2 sessions/week when the goal is greater body-shape improvement; effects were comparable in boys and girls.

Future studies should include larger, multi-site samples, longer intervention periods, and post-intervention follow-up to determine the minimum effective frequency required both to induce and maintain benefits. Objective assessment of diet, habitual physical activity, sedentary behavior, and exercise load should be incorporated to clarify mechanisms underlying the observed frequency-response patterns. In addition, future trials should use gold-standard assessments, such as direct cardiopulmonary exercise testing and DXA- or imaging-based body composition measures, and should examine whether age, sex, and biological maturation modify responsiveness to school-based HIIT. Prior school-based HIIT research suggests that indicators such as age at peak height velocity may influence CRF adaptations and may interact with sex.^37^ Maintenance-phase and implementation studies are also needed to identify scalable strategies for sustaining adherence and long-term effectiveness in school settings.

### Implications

#### Possible mechanisms underlying diminishing returns at higher weekly frequency

The modest incremental gains from two to three HIIT sessions/week may reflect several physiological and behavioral factors. First, early-phase adaptations in previously inactive children may be rapid, with subsequent sessions yielding smaller marginal gains as aerobic and neuromuscular efficiency improves.^38^ Second, when frequency increases without proportional increases in recovery capacity, residual fatigue may blunt the quality of high-intensity efforts, thereby reducing the “effective” stimulus per session.^39^ Third, metabolic and hormonal responses to repeated high-intensity bouts (e.g., transient improvements in insulin sensitivity, catecholamine-mediated lipolysis, appetite regulation, and substrate utilization) may show non-linear dose-response characteristics, with diminishing additional benefit once a threshold of weekly vigorous activity is achieved.^40^^,^^41^ Finally, behavioral compensation (e.g., reduced spontaneous activity outside training) is possible when exercise frequency increases, which could attenuate net energy balance and thus BMI-related outcomes. Collectively, these considerations support the concept of a minimum effective weekly dose for some outcomes, with higher frequencies primarily beneficial when recovery and overall activity patterns are maintained.

In broader exercise-nutrition research, antioxidant or plant-derived supplementation combined with high-intensity training has been reported to influence adipokines and inflammatory markers; however, because these studies largely involve adult samples and co-interventions, their relevance to PE-embedded HIIT in children is indirect and should be interpreted cautiously.^42^^,^^43^^,^^44^^,^^45^^,^^46^^,^^47^

#### Influence of diet and total physical activity on observed dose-response patterns

Dietary intake was partially standardized during school days (breakfast and lunch were provided at school according to national dietary guidance), but dinner and overall activity outside supervised PE were not objectively monitored, which may have introduced variability into both adiposity and fitness outcomes. Even modest changes in caloric intake, snacking behavior, or meal regularity can meaningfully influence short-term body-composition trajectories,^48^ potentially amplifying or masking frequency-dependent training effects. Likewise, unmeasured extracurricular physical activity and sedentary time (including screen time) could confound interpretation of dose-response relationships: participants with higher prescribed HIIT frequency might compensate by reducing habitual activity,^49^ whereas others may increase overall activity due to improved fitness or motivation. Therefore, the observed frequency-response patterns should be interpreted cautiously as effects of prescribed HIIT frequency within a real-world school context rather than isolated physiological effects of weekly exercise load. Future trials should incorporate standardized dietary assessment and objective monitoring of total physical activity and sedentary behavior to strengthen causal attribution and clarify mechanisms.

#### Practical implementation in school settings

From a translational perspective, the protocol is feasible because it can be embedded within existing PE lessons with minimal equipment and space. In our setting, the structured HIIT segment (warm-up, HIIT, and cool-down) occupied ∼16 min and was delivered at the end of a standard 40-min PE class, allowing the remaining time to follow the school curriculum. For schools facing time constraints, a once-weekly “entry dose” may be an acceptable starting point to improve aerobic fitness and adiposity while minimizing disruption to teaching plans. Where resources allow, progressing to two sessions per week may maximize returns for BMI-related outcomes. Scaling such programs may require brief staff training (interval timing, pacing cues, class management, and safety), standardized lesson scripts, and engagement strategies (team-based challenges, feedback on effort, and age-appropriate progression). These considerations support flexible, PE-embedded HIIT as a scalable option for pediatric obesity management in real-world school systems.

#### Sustainability and maintenance of benefits

Whether the observed improvements persist after a supervised 12-week program is uncertain. In children and adolescents, detraining and seasonal changes in school routines can lead to partial loss of fitness and attenuation of body-composition improvements if vigorous activity is not maintained. A practical approach may be to transition to a maintenance phase (e.g., one HIIT session per week integrated into PE) after an initial higher-frequency period, combined with periodic variation in exercises to sustain motivation. Incorporating variety (rotating movement patterns), gamification (points, teamwork, short-term goals), and autonomy-supportive teaching may enhance enjoyment and adherence, which are key determinants of long-term participation. Future studies should include post-intervention follow-up and evaluate maintenance strategies to determine the minimal dose required to preserve gains.

### Limitations of the study

This study has several limitations. First, the sample size was modest and drawn from a single region, which may limit generalizability and reduce power to detect small effects, particularly for secondary outcomes and sex-specific differences. Second, the 12-week intervention may have been insufficient to reveal longer-term divergence across HIIT frequencies or the durability of adaptations after the program ended. Third, dietary intake and physical activity outside the supervised PE sessions were not objectively monitored, which may have influenced both adiposity- and fitness-related outcomes. Fourth, biological maturation was not quantified beyond Tanner stage screening and was not modeled analytically, so potential moderating effects of maturity status cannot be excluded. Finally, CRF was assessed using 20mSRT performance and 20mSRT-derived estimated VO_2_max rather than direct cardiopulmonary exercise testing. Recent methodological work has cautioned against interpreting 20mSRT-derived estimates as true VO_2_max in children, and such estimates may vary according to the prediction equation and testing conditions.^50^^,^^51^ Accordingly, the present findings are best interpreted as improvements in shuttle-run performance and field-estimated aerobic fitness rather than directly measured VO_2_max.

## Resource availability

### Lead contact

Further information and requests for resources should be directed to and will be fulfilled by the lead contact, Meng Cao (caomengsus@163.com).

### Materials availability

This study did not generate new unique reagents or materials.

### Data and code availability

• Data: The datasets generated and analyzed during the current study are available from the lead contact upon reasonable request.
• Code: This study did not generate or use custom code.
• Other: Any additional information required to reanalyze the data reported in this paper is available from the lead contact upon request.

## Acknowledgments

We are grateful to the participating students and their families, as well as the partnering schools and physical education teachers for their cooperation during testing and training sessions. We also thank the pediatric consultants and school administrators for facilitating screening and safety oversight and the research assistants who supported data collection and quality control. This study was supported by the Futian Healthcare Research Project (No. FTWS109 and No. FTWS2025074).

## Author contributions

Y.G.: conceptualization, methodology, investigation, formal analysis, data curation, visualization, writing – original draft.; M.C.: conceptualization, methodology, supervision, project administration, writing – review and editing, correspondence.; Y.X.: resources, clinical coordination, participant recruitment and testing coordination, data validation, funding acquisition, writing – review and editing, correspondence.; Y.Z.: investigation, data curation.; T.Z.: data validation, writing – review and editing.; C.W.: formal analysis, writing – review and editing.; X.W.: investigation, resources.; All authors contributed to the article, read and approved the final manuscript, and agree to be accountable for all aspects of the work. M.C. served as the lead contact.

## Declaration of interests

The authors declare no competing interests.

## STAR★Methods

### Key resources table

**Table:** 

REAGENT or RESOURCE	SOURCE	IDENTIFIER
**Software and algorithms**		

IBM SPSS Statistics 25.0	IBM Corp.	RRID:SCR_002865
G∗Power 3.1	Heinrich Heine University Düsseldorf	RRID:SCR_013726
RStudio 2024.12.1	Posit Software	RRID:SCR_000432

**Other**		

InBody770 Body Composition Analyzer	InBody Co., Seoul, Korea	Model: InBody770
RGZ-120-RT Stadiometer	Wuxi Weighing Apparatus Factory Co., Ltd., Wuxi, China	Model: RGZ-120-RT
Polar Team OH1 Heart Rate Monitor	Polar Electro Oy, Finland	Model: OH1
Omron HEM-1020 Blood Pressure Monitor	Omron Healthcare, Osaka, Japan	Model: HEM-1020
Casio Stopwatch	Casio Computer Co., Ltd., Japan	N/A
20 m Shuttle Run Test Protocol	See Reference: Léger et al., 1988	https://doi.org/10.1080/02640418808729800
Dataset generated in this study	This paper	Available from the lead contact upon reasonable request

### Experimental model and study participant details

This study was a 12-week, four-arm, parallel-group randomized controlled trial conducted in a school setting, with a 2-week baseline lead-in period. The trial adhered to the CONSORT guidelines for randomized trials and to the Declaration of Helsinki. Ethics approval was granted by the Medical Ethics Committee of Shenzhen University (PN-2020-045), and the trial was prospectively registered in the Chinese Clinical Trial Registry (ChiCTR2100048737). Written informed consent was obtained from participants and their legal guardians, and written assent was obtained from all minors prior to participation. Outcome assessors and data analysts were masked to group allocation.

Participants were children aged 10–12 years with obesity, recruited through school-based screening in collaboration with pediatricians. Eligibility criteria are presented in the table below. In brief, participants were eligible if they were aged 10–12 years, met the criterion for obesity according to BMI ≥95th percentile, were enrolled in regular school physical education (PE) classes, and were classified as Tanner stage II by the school physician at enrollment. Exclusion criteria included any contraindication to vigorous exercise, current use of medications known to affect metabolism or growth, participation in structured athletic training outside school during the study period, or inability to complete baseline or follow-up assessments. Eligibility was reviewed by the school physician, head teacher, and PE teacher prior to participation.

**Table:** Eligibility criteria of the study

Inclusion criteria	Exclusion criteria
• Aged 10–12 years. • Tanner stage II (assessed by a school physician). • BMI ≥95th percentile for gender and age. • Free of any medication that could interact with the protocol (e.g., cardiac, orthopedic, neuromuscular, or neurological disorders). • No contraindication to physical activity. • Self-reported physical activity<2 h/week (IPAQ).	• history of major cardiovascular, metabolic, or neurological diseases. • use of medication affecting exercise or metabolism. • metal or electrical prostheses. • participation in another weight-loss or exercise program. • unable to understand or follow the protocol. • deemed unsuitable by investigators.

BMI, body mass index; IPAQ, International Physical Activity Questionnaire.

**Figure 2:**
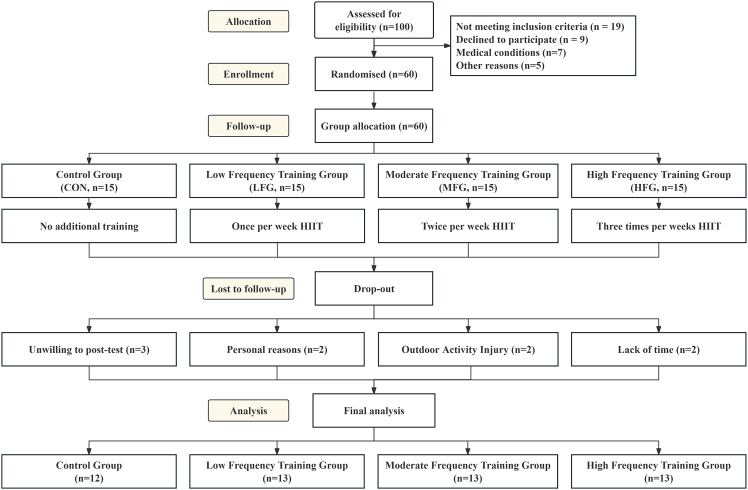
CONSORT flow diagram of participant recruitment, allocation, follow-up, and analysis The diagram shows participant screening, randomization, follow-up, and analysis across the four study groups.

**Figure 3:**
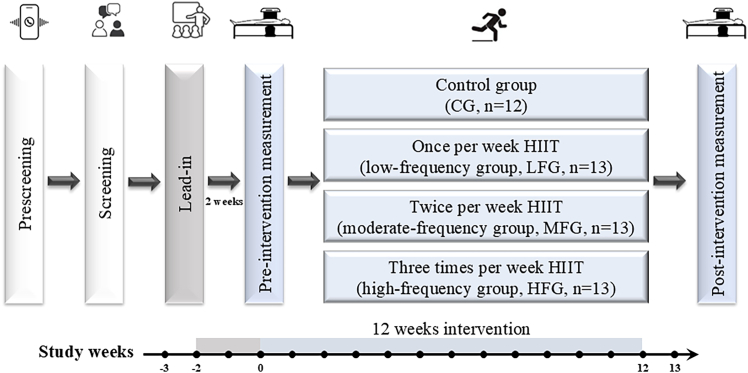
Study design Pre-screening of potential participants was performed by phone calls, and eligible participants were scheduled for a screening visit. The randomized controlled trial included a 2-week baseline (lead-in period) and a 12-week intervention.

A total of 60 participants were randomized equally across four groups (n = 15 per group). Fifty-one participants completed the post-intervention assessments (control group [CG] = 12; low-frequency group [LFG] = 13; moderate-frequency group [MFG] = 13; high-frequency group [HFG] = 13), corresponding to an overall completion rate of 85%. Participant flow is shown in Figure 2, and the overall study design is shown in Figure 3. Nine participants did not complete post-testing because of unwillingness to attend the post-test (n = 3), personal reasons (n = 2), lack of time (n = 2), and injuries sustained during non-study outdoor activities (n = 2).

### Method details

#### Intervention protocol

The intervention was delivered during regular PE classes on the school campus rather than as an extracurricular program. Each PE class lasted 40 min and followed the school’s standardized PE curriculum. The HIIT protocol was embedded in the final segment of the PE lesson. Participants assigned to the intervention completed the HIIT segment 1, 2, or 3 times per week, depending on group allocation, on non-consecutive days whenever possible. The remainder of each PE class followed the same school curriculum.

Each 40-min PE lesson comprised two parts: (1) a curriculum-based PE component of approximately 24 min delivered according to the school’s unified teaching plan, including ball skills, fundamental movement skills, submaximal running drills, and class coordination tasks; and (2) a structured HIIT component of approximately 16 min implemented at the end of the class, consisting of a 5-min standardized warm-up, 6-min HIIT, and 5-min cool-down. Apart from replacing the final approximately 16 min with the HIIT segment, no additional exercise was prescribed for the intervention groups. The school required the curriculum-based PE component to remain similar across classes and grade-level groups, and the same PE teachers delivered the curriculum component across groups to reduce between-class differences.

**Figure 4:**
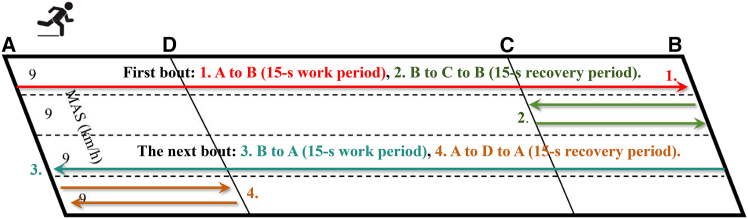
Schematic of the HIIT run protocol The schematic illustrates the running-based HIIT protocol, including 15-s work intervals, 15-s recovery intervals, and pacing based on individualized target distances.

The running-based HIIT protocol was performed on the school track or field. Pacing was implemented using cones and track markings indicating individualized target distances corresponding to 100% and 50% of maximal aerobic speed (MAS). A Casio stopwatch was used to time the 15-s work and 15-s recovery intervals. After each 15-s sprint, participants returned to a common start line during the recovery period to maintain order and synchronize interval timing for the next repetition. A schematic of the protocol is shown in Figure 4.

Attendance was recorded by PE teachers at each session. If a participant missed a scheduled HIIT session because of absence, a supervised make-up session was arranged after school on the same day or the following day. The *a priori* adherence criterion was ≥90% attendance, and adherence remained ≥90% after make-up sessions.

Participants were recruited shortly after the routine school health examination, and eligibility was reviewed by the school physician, head teacher, and PE teacher before participation. During training, any discomfort was evaluated immediately by the school physician and on-site medical personnel. Two participants in the twice-weekly group withdrew after a non-training incident involving an ankle sprain while walking downstairs after class; this is documented in the participant flow diagram.

Participants allocated to the control group continued the school’s usual PE curriculum throughout the 12-week period, with three 40-min classes per week, and did not receive the structured HIIT segment. Each control-group PE class was delivered by school PE teachers according to the grade-level teaching plan and typically consisted of a brief warm-up, curriculum-based skill practice and games, and cool-down or organization. These classes did not include any prespecified interval format, such as repeated maximal 15-s efforts with timed recoveries, or other research-led high-intensity components.

The intended intensity of the school PE curriculum was predominantly moderate and below 80% of age-predicted maximal heart rate according to school teaching requirements. To minimize contamination, all participants were instructed to maintain their usual lifestyle and not begin any additional structured training during the study period. Reminders were delivered to families through the parent communication group by school staff.

Training intensity during the HIIT segment was monitored in real time using the Polar Team system (Polar Team, Finland) for safety supervision and to support session fidelity. The on-site coach or PE teacher viewed live heart-rate displays and provided immediate cues, such as “speed up,” “maintain,” or “slow down,” to help participants align effort with the target vigorous-intensity zone of 80%–90% of age-predicted maximal heart rate during work intervals. Rating of perceived exertion was not collected. Because Polar Team session logs were not exported and retained in an analyzable format at the time of the trial, objective heart-rate and RPE metrics are not reported and should be considered a limitation.

#### Anthropometry and body composition

Participants were required to fast for 10 h prior to measurement. During assessments, they removed their shoes and wore lightweight clothing. Standing height was measured to the nearest 0.1 cm using a stadiometer (RGZ-120-RT, Wuxi Weighing Apparatus Factory Co., Ltd., Wuxi, China), with participants barefoot and upright according to standardized anthropometric procedures. Body mass, fat mass, body fat percentage (BF%), lean body mass, and visceral adipose tissue (VAT; BIA-derived estimate) were assessed using a bioelectrical impedance analyzer (InBody770, InBody Co., Seoul, Korea). The analyzer was calibrated before use.

Because BIA-derived indices, including VAT estimates, are not gold-standard measures, assessments were conducted under standardized pre-assessment conditions, including overnight fasting, lightweight clothing, and removal of metallic accessories, and were performed using the same device and protocol by trained staff to reduce measurement variability.

Body mass index (BMI) was calculated as body weight in kilograms divided by height in meters squared. Age- and sex-adjusted BMI z-scores (BMI-z) were derived using the 2007 World Health Organization growth reference for school-aged youth, based on the LMS method.^52^ BMI-z represents the number of standard deviations an individual’s BMI differs from the mean BMI of the reference population of the same age and sex.

(Equation 1) $$BMI\;z-score=\frac{{\left(BMI/M\right)}^{L}-\;1}{\left(L\times S\right)}\;if\;L\neq 0$$

(Equation 2) $$BMI\;z-score=\frac{\ln\left(BMI/M\right)}{S}\;if\;L=0$$

#### Cardiorespiratory fitness

Cardiorespiratory fitness (CRF) and maximal aerobic speed were assessed using the 20-m shuttle run test (20mSRT), a widely used field-based method for estimating aerobic capacity in youth.^53^^,^^54^ The test was performed on a school track, with participants running repeatedly between two lines 20 m apart while following auditory signals that progressively increased in frequency. The protocol started at 8.5 km/h and increased by 0.5 km/h each minute. Testing ended when participants failed to reach the line on two consecutive occasions or voluntarily stopped. Maximal aerobic speed was determined from the final completed stage. Heart rate was monitored continuously using a wireless device (Polar Team OH1, Finland) to support safety monitoring and encourage maximal effort.^55^

The 20mSRT was administered at baseline and after the 12-week intervention. Testing took place between 16:00 and 17:00, with 2–8 participants assessed per session. Three trained teachers supervised the procedure, and one researcher recorded results. Estimated VO_2_max (mL·kg^-1^·min^-1^) was calculated from 20mSRT performance using the equation proposed by Mahar et al.^56^

(Equation 3) $$VO_{2}\;\max=41.76799+\left(0.49261\times \text{laps}\right)-\left(0.00290\times {\text{laps}}^{2}\right)-\left(0.061613\times \text{BMI}\right)+\left(0.34787\times \text{gender}\times \text{age}\right)$$

#### Other clinical measures

Waist circumference (WC, cm) was measured at the midpoint between the lowest rib and the iliac crest using a non-elastic tape. Measurements were taken at the end of a normal exhalation. Two measurements were obtained, and the mean value was used for analysis.

Resting blood pressure (BP, mmHg) was measured after 10 min of seated rest using an automated sphygmomanometer (Omron HEM-1020, Osaka, Japan). The cuff was placed on the left upper arm at heart level. Two readings were recorded 1 min apart and averaged for analysis.

#### Dietary context

No dietary intervention was implemented as part of the trial. To minimize major dietary fluctuations during the study, parents or guardians received routine nutrition education delivered by a hospital dietitian, including practical guidance for preparing balanced breakfasts and dinners. In addition, students’ breakfast and lunch were provided on campus through a standardized school meal program with unified menus, portion sizes, and energy targets, and relevant meal records were available for traceability. Throughout the intervention, the school physician sent daily reminders to families to encourage adherence to usual eating routines and to follow the provided dietary guidance. However, individual-level dietary intake, including total energy and macronutrient composition, was not recorded or quantified; therefore, residual dietary variability cannot be excluded.

### Quantification and statistical analysis

#### Sample size estimation

*A priori* sample-size estimation was conducted for the two prespecified primary outcomes, BMI-z and 20mSRT performance. Based on previous school-based HIIT trials reporting a moderate effect on 20mSRT outcomes (standardized effect size g = 0.60; corresponding ANOVA effect size f = 0.30), a four-arm parallel design with two-sided α = 0.05 and 80% power in G∗Power 3.1 required a minimum of 52 participants, corresponding to approximately 13 participants per group. To account for potential attrition of approximately 15%, the recruitment target was set at 60 participants (15 per group). Sample-size estimates for BMI-z under similar assumptions did not exceed this threshold, so the larger estimate was adopted. Ultimately, 60 participants were randomized.

Post hoc power estimates can be difficult to interpret because they depend on the observed effect size; therefore, a sensitivity analysis was additionally considered. Given the final sample of 51 participants across four groups and two-sided α = 0.05, the study had 80% power to detect a moderate-to-large between-group effect of approximately f = 0.48 under a conservative one-way ANOVA model. Accordingly, the trial was more likely to detect moderate-to-large effects, whereas small effects, particularly for some secondary outcomes, may have been underpowered.

#### Statistical analysis

Statistical analyses were performed using SPSS Statistics version 25.0 (IBM, Armonk, NY, USA). Data are presented as mean ± SD. The prespecified primary outcomes were BMI-z and 20mSRT performance, and the significance threshold for primary analyses was two-tailed α = 0.05. The 20mSRT-derived estimated VO_2_max was reported as a supportive outcome and interpreted cautiously. Secondary outcomes, including BMI, BF%, fat mass, and waist-to-hip ratio (WHR), were evaluated using a Bonferroni-adjusted α of 0.0125 (0.05/4). Estimated VAT, fat-free mass (FFM), systolic blood pressure (SBP), and diastolic blood pressure (DBP) were treated as exploratory outcomes.

Model assumptions were evaluated before analysis. Normality of residuals was assessed using the Shapiro–Wilk test, homogeneity of variances using Levene’s test, and sphericity for repeated-measures ANOVA using Mauchly’s test, with Greenhouse–Geisser correction applied where necessary. For ANCOVA models, linearity and homogeneity of regression slopes were also examined.

Time and group effects were assessed using repeated-measures ANOVA with Bonferroni-adjusted post hoc comparisons where appropriate. Mean differences in change scores (Δ) and 95% confidence intervals are reported. Because baseline 20mSRT showed a chance imbalance, ANCOVA models adjusting for baseline values were used for relevant between-group follow-up comparisons. Dose–response relationships among the HIIT groups (1, 2, and 3 sessions per week) were evaluated using polynomial regression, comparing linear and quadratic models using ΔR^2^ and Akaike information criterion. Effect sizes are reported as partial η^2^.
